# Gold, Silver, and Palladium Nanoparticles: A Chemical Tool for Biomedical Applications

**DOI:** 10.3389/fchem.2020.00376

**Published:** 2020-06-03

**Authors:** Sundas Bahar Yaqoob, Rohana Adnan, Raja Muhammad Rameez Khan, Mohammad Rashid

**Affiliations:** ^1^Department of Zoology, Mirpur University of Science and Technology Mirpur, Mirpur, Pakistan; ^2^School of Chemical Sciences, Universiti Sains Malaysia, Penang, Malaysia; ^3^Department of Chemistry, Mirpur University of Science and Technology Mirpur, Mirpur, Pakistan

**Keywords:** nanoparticles, gold, silver, palladium, toxicity mechanism, physico-chemical toxicity

## Abstract

Herein, the role of metal-based nanoparticles (NPs) in biomedical analysis and the treatment of critical deceases been highlighted. In the world of nanotechnology, noble elements such as the gold/silver/palladium (Au/Ag/Pd) NPs are the most promising emerging trend to design bioengineering materials that could to be employed as modern diagnostic tools and devices to combat serious diseases. NPs are considered a powerful and advanced chemical tool to diagnose and to cure critical ailments such as HIV, cancer, and other types of infectious illnesses. The treatment of cancer is the most significant application of nanotechnology which is based on premature tumor detection and analysis of cancer cells through Nano-devices. The fascinating characteristic properties of NPs—such as high surface area, high surface Plasmon resonance, multi-functionalization, highly stable nature, and easy processing—make them more prolific for nanotechnology. In this review article, the multifunctional roles of Au/Ag/Pd NPs in the field of medical science, the physicochemical toxicity dependent properties, and the interaction mechanism is highlighted. Due to the cytotoxicity of Ag/Au/Pd NPs, the conclusion and future remarks emphasize the need for further research to minimize the toxicity of NPs in the bio-medicinal field.

## Introduction

The advent ofproduction of large advanced Nano-scaled technology encourages the usage of noble metal-based NPs for diagnosis of diseases, selected drug delivery, biological treatment of cancerous cells, and bio-imaging. The NPs' size, optoelectronic properties, and structural morphology has attracted the attention of researchers toward their potential biomedical applications (Zhang et al., 2019). There has been progress in the synthesis of Nano-sized materials and the surface engineering on these particles through definite ligands that allow the NPs to be modified to achieve the precise shape, biocompatibility, disparity (Rai et al., 2016). This bioengineering modification permitted the development of the drugs that are based on metal architectures framework. However, the silver, palladium, gold, and iron nanostructures are widely studied in modern research. The vastly complex, multi-layered and multifactorial connections of metal-based nanoparticles (NPs) with biotic systems are of great importance. The absence of the stability of toxicological facts prevent the extensive application of these Nano-based pharmaceuticals (Bierkandt et al., 2018; Azharuddin et al., 2019). Moreover, constant efforts have been made to improve their activities and safety profiles for clinical applications. The NPs acquire potential characteristics and features e.g., molecular fluorophores which are efficient to make an ideal source for diagnostic-based applications (Agarwal et al., 2019; Khadpekar et al., 2019). The characteristic properties of NPs are: the size must be in the rage of 1–100 nm and have great surface-volume relation; shape and compositional requirements; qualitative and quantitative target-binding activities; and the greater toughness (Kumar et al., 2011; Sharma et al., 2019; Singh et al., 2019). The size of NPs plays an important role in Nano-biotechnology that makes it more prolific for all of their biological applications. Nano-range proposes several more benefits than other bulk structure materials. Nanoparticles' chemical and physical characteristics can have a substantial effect on general functions like target-binding activities. The physiochemical properties of NPs, increase the tolerance toward biocompatibility. In the biological sciences, NPs have an extensive history of bio-conjugation with protein, peptides and DNA, cellular delivery systems and labeling agents (Leso and Iavicoli, 2018). The light-scattering property of the material depends on the shape, size, and surface of the molecule, which makes the material prolific for multiplexed agent exposure for biotic moieties. Currently, several methods and technologies for engineered-based materials like superficial modification, size control, and designing have touched upon the advance production of large Nano-range NPs for both micro and macro size molecules (Singh et al., 2018; Handoko et al., 2019; Yaqoob et al., 2020c). According to the literature, engineered NPs with abilities to modify physical and chemical characteristics, offer to make it more progressive and extremely vigorous in terms of binding affinities for several types of biomolecules and the treatment of cancerous cells and viruses like HIV. In brief, the most significant breakthroughs in today's modern chemical sciences are attributed to nanotechnology (Aderibigbe, 2017). Mostly, the noble metals are considered useful due to notable catalytic, electronic, magnetic, optical, and mechanical properties. The characteristics and possible applications of nano range noble metals have developed a significant and theoretical approach in different applications (Sakthivel et al., 2019). In this regard, the most commonly employed noble metals are Au, Ag, and Pd that are very rare and valuable. These carried a great revolution in the biological and medicinal fields in the modern era as compared to other materials (Yaqoob et al., 2020a). The unusual physical property of the gold metal such as high resistance to corrosion makes it bio-compatible for medical applications; it is very suitable for dealing with a diversity of diseases like skin ulcers, syphilis, cancer, smallpox, AIDS and measles (Daniel and Astruc, 2004; Chitra, 2018). Currently, gold nanoparticles (AuNPs) received much attention for the critical disease diagnosis and the treatment of several diseases e.g., cancer, HIV, and rheumatoid arthritis, whereas substantial exploration is currently promising for possible applications as anticancer, antibacterial and bio-diagnostic material (Yaqoob et al., 2019). AuNPs contain substantial positions in the medical treatment field due to their relative chemical stability that makes the preparation and fabrication methods modest, straightforward, and less dangerous. Additionally, the developments led the scientists to produce Nanostructures that can be conjugated to numerous types of biotic molecules with antibodies (Shenoy et al., 2005; Jabr-Milane et al., 2008; Ho et al., 2010). It can influence the targeted cells stating through receptors. Colloidal-based Au has been carrying a significant role in the medical field while the precise mechanism to exploit it is not completely understood. Nowadays, the AuNP's applications are growing gradually in pharmacological sciences for human safety. It can be employed to comprehend further about diseases such as HIV and cancer by offering substantial targets through Nano-vesicles (Potineni et al., 2003). Silver (Ag) has been used such as an anti-microbial, anti-viral e.g., Phoenicians employed silver vessels to preserve wine and water throughout their far-reaching voyages. In the earlier time, ancient Egyptians thought that the Ag metal is valuable for healing disease (Likus et al., 2013; Yaqoob et al., 2020b). Therefore, AgNP-based chemicals are utilized for the elimination of wound infection before antibiotics. Nowadays, Ag-based cream named Ag-sulfadiazine is commonly used for antibacterial treatment to cure serious wounds (Aziz et al., 2019). However, insufficient local retention and cytotoxic influence limited the experimental utilization of Ag material. Further, the significant magnetic, transparent and electronic properties of AgNPs can be applied to control the microbial activities (Murphy et al., 2015). Another noble metal is palladium (Pd) that has extraordinary catalytic, and electronic features (Chaloupka et al., 2010; Pattadar et al., 2019). Palladium (Pd)NPs structures have been extensively used in applications such as the electrical equipment composition, as a sensor for exposure to several bio-analytes. In biomedicine, Pd is very frequently used in dentistry appliances and especially its needles are mostly applied in the health center for prostate cancer therapy and choroidal melanoma therapies. So, despite their notable properties, PdNPs have not been used, until lately, in the Nano-biomedicine applications and the PdNPs offer the prospect for the presence of further active catalyst resources due to presence of larger surface-area-to-volume ratio and its greater surface energy values make it more inexhaustible in the remedial field (Bakuru et al., 2018; Leso and Iavicoli, 2018). In this article, the focus is on the application of noble NP metals, especially, Au, Ag, and Pd in the analysis and bioremediation of diseases and the level of their toxicity. The toxicity of the NPs is a very important aspect in the medical applications, herein, we have critically analyzed the physical and chemical properties associated with their toxic effect. Finally, a future perspective of Au, Ag and Pd and the mechanism of toxicity are summarized which might be useful for future research, since these three noble metals have great potential in upcoming biomedical research.

## Au/Ag/PdNPs-A Biomedical Perspective

Disease treatment is hindered through drug resistance, demonstrating an urgent need to introduce novel therapeutics agents. The scientific community declared the metallic-based Au/Ag/PdNPs have been developed for several diseases to remediate effectively. Au/Ag/PdNPs are categorized by their nano range of 10–100 nm. They are considered useful due to the unique interaction of molecules within or outside the cell surface. The high surface area of these particles indicates better cell permeability which also refers to the meaningful outcomes. However, the noble metallic NPs contain excellent physical/ chemical and surface charge properties. These kinds of exclusive properties prefer the metallic NPs, especially Au/Ag/Pd as a potential and significant therapeutic agent for biological treatment (Rai et al., 2013). Metallic NPs (Au/Ag/Pd) are considered as a potential source for biomedical applications which contain the most effective properties such as highly stable aggregate activities, non-toxic, biocompatible, specific to target cells and tissues and very easily available. A brief outline of Au/Ag/Pd is summarized below.

## Gold Nanoparticles

### Cancer, Antibacterial, and HIV Treatment

**Figure 1:**
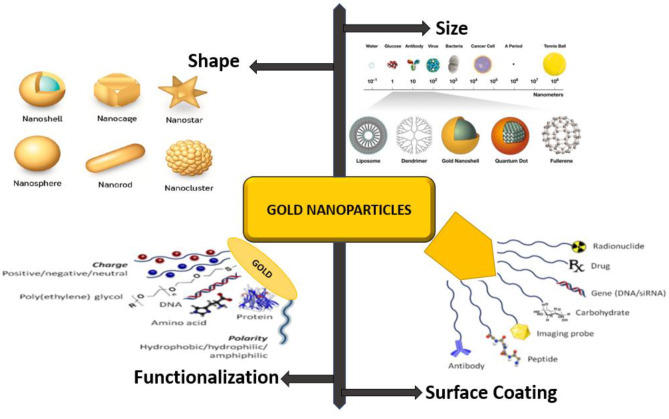
Spherical to Nanostar morphology of AuNPs, Functionalization by Antibody, Carbohydrate, DNA/RNA, peptide, loading with drugs, and used for probing Image.

The AuNPs have received much attention due to their benchmark properties. They have been studied as a potential source for the treatment and diagnosis of cancerous cells, anti-bacterial activity, AIDS, drug delivery, and bioimaging therapy. Furthermore, the non-immunogenic and non-toxic nature of AuNPs with high permeability and the high retention effect suggest further support of the informal accumulation and penetration for the treatment of tumor sites. Several advanced methods of AuNPs are still under development due to its distinctive values in the therapeutic field (Kumar et al., 2011; Di-Gianvincenzo et al., 2012; Her et al., 2017; Kim, 2019). AuNPs formerly played a substantial part in human welfare in the clinical diagnostic field as well as numerous medical applications. Further advanced research demonstrates that AuNPs are becoming attractive and the most promising method in cancer, anti-bacterial and AIDS treatment. The shape, size, surface coating, functionalization, and interaction of AuNps has been demonstrated in Figure 1. However, the application of AuNPs in the advancement of nanotechnology is the most incredible and highly promising in the treatment of cancer cells.

**Figure 2:**
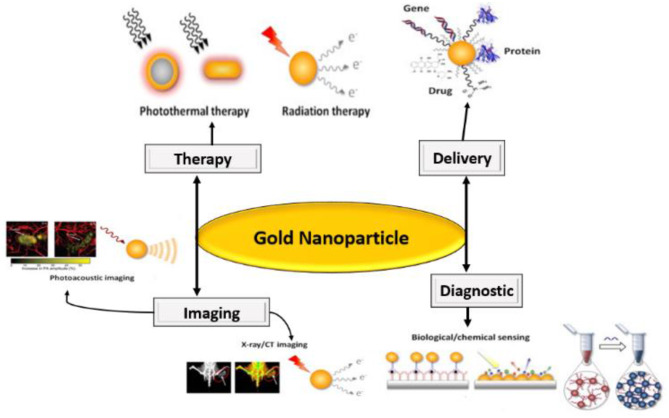
Application of AuNPs in medicinal science.

The AuNPs applications in various biomedical techniques such as X-rays/ CT bioimaging, photoacoustic imaging, chemical sensing, photothermal, radiation therapy and drugs delivery has also been studied as shown in Figure 2.

AuNPs have been stated as the most active antibacterial agents as shown in the literature, Table 1. The green synthesized AuNPs actively work against *Corynebacterium pseudotuberculosis*, these bacteria cause a serious bacterial infection which is commonly found in sheep (Huang et al., 2011a; Mohamed et al., 2017). The antibacterial activities of AuNPs are significant due to the better penetration ability through the cell wall of the target body. Zhou et al. (2012) stated that AuNPs suppressed *E. coli* growth. Li et al. (2014) studied the functionalized AuNPs as a most efficient and active medium against gram-negative and gram-positive multi-drug resistance pathogens. The cationic hydrophobic AuNPs were active toward the bacterial cell membrane integrity and produced instability in the bacterial cells. AuNPs showed a slightly toxic effect on the mammalian body cells. Antibacterial activities of AuNPs against gram-positive and negative bacteria are not similar due to bacterial membrane structure which is recommended for the high dose of NPs. AuNPs antibacterial activities occur due to the condensed adenosine triphosphate synthase activity that disturbs the basic process of metabolism and distorts the ribosome units for tRNA binding, therefore, a failure of bacterial biotic mechanism occurs. The large surface area of AuNPs improves the interaction with bacterial cells (Shamaila et al., 2016). The AuNPs affect bacterial cytoplasm and protein that cause cell death. AuNPs, when combined with antibiotics, offered enhanced antibacterial activities. Certain antibiotics have been employed such as kanamycin, gentamicin, vancomycin, ampicillin, streptomycin, and levofloxacin. The AuNPs did not show antibacterial activities, though they served as a drug transporter for gentamicin distribution due to their large surface area (Dasari et al., 2015). The antibacterial activities of AuNPs were not substantial when likened with gentamicin. Saha et al. (2007) collectively studied AuNPs with kanamycin, streptomycin, and ampicillin. The combinations were quite active against *Micrococcus luteus, E. coli*, and *Staphylococcus aureus*. AuNPs, when shared with kanamycin and *streptomycin* presented considerable antibacterial activities. The insignificant antibacterial activities of AuNPs along with ampicillin has exposed poor stability, though the free AuNPs did not show antibacterial activities. AuNPs when not combined with antibiotics is unproductive against powerful bacteria. Therefore, they serve as medication carriers and their mixture with antibiotics results in synergistic antibacterial actions. AuNPs are steady and their capacity to interrelate with bacterial cells empowers them as a significant antibacterial agent (Santhoshkumar et al., 2014).

**Table 1:** AuNPs antibacterial activity with their therapeutic influence.

**Metal nanoparticles**	**Type of bacteria**	**Therapeutic effect**	**References**
AuNPs	*Corynebacterium pseudotuberculosis*	Effectively work against bacterial infection	Shaikh et al., 2019
AuNPs	*E. coli*	Actively work against *E. coli*	Zhou et al., 2012
AuNPs	*E. coli*	NPs were vigorous against Gram- positive, Gram- negative multi-drug resistant pathogens	Li et al., 2014
AuNPs	*E. coli, Staphylococcus aureus, Bacillus subtilis* and *Klebsiella pneumonia*	Active against bacterial infection	Shamaila et al., 2016
AuNPs	*E. coli, S. typhimurium DT104*, and *S. aureus*	Actively work against bacterial infection	Dasari et al., 2015
AuNPs	*Salmonella typhi*	Actively inhibited the bacterial growth	Lima et al., 2013
AuNPs	*Staphylococcus aureus and E. coli*	Active against *Staphylococcus aureus* and *Escherichia coli*	MubarakAli et al., 2011
AuNPs shared properties with ampicillin	*E. coli, Micrococcus luteus* and *Staphylococcus aureus*	Actively work against the bacteria	Saha et al., 2007
AuNPs collective properties with streptomycin	*E. coli, Micrococcus luteus* and *Staphylococcus aureus*	Potentially work against the bacteria	Saha et al., 2007
AuNPs shared properties with kanamycin	*E. coli, Micrococcus luteus* and *Staphylococcus aureus*	Potentially work against the bacteria	Saha et al., 2007
AuNPs collective properties with levofloxacin	*S. aureus* and *E. coli*	Inhibited the bacterial growth	Bagga et al., 2016
AuNPs shared properties with vancomycin	-	Selectively bind the cells of Gram-positive and negative Bacteria, Antibiotic-resistant bacteria	Gu et al., 2003
Light-absorbing AuNPs conjugated with precise antibodies	*Staphylococcus aureus*	Selective killing of bacterium	Zharov et al., 2006
AuNPs shared properties covered with cefaclor	*Staphylococcus aureus, E. coli*	Effective antibacterial activities against Gram+ive and Gram-tive bacteria	Zhang et al., 2015
AuNPs shared properties with gentamicin	Active against *Escherichia coli*		Rai et al., 2019
AuNPs combined with gentamicin	-	Excellent antibacterial activities	Burygin et al., 2009
AuNPs synthesize by using *Stoechospermum marginatum*	-	Improved antibacterial activities	Rajathi et al., 2012
AuNPs synthesize through using aqueous moringa oleifera leaves	*Staphylococcus aureus*	Effective against bacterium	Prasad and Elumalai, 2011
AuNPs synthesize from A. comosus extract	-	Useful decontamination methods for inhibiting the bacterial growth	Bindhu and Umadevi, 2014
AuNPs Synthesize by using with ofloxacin	-	Greater bactericidal property	Ahmed et al., 2014
AuNPs Synthesize by using banana peel extract	-	Improved antibacterial activity	Bankar et al., 2010

**Figure 3:**
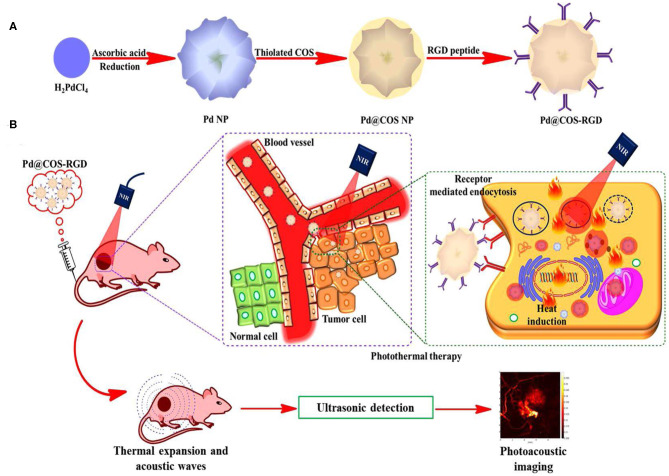
**(A)** A systematic representation of preparation of PdNPs, functionalized by surface coating of thiolate chitosan oligosaccharide Pd@COS NPs and followed by functionalization with RGD peptide. **(B)** A schematic representation of Pd@COS-RGD showing the thotothermal ablation and photoacoustic imaging at tumor site [Adapted from (Bharathiraja et al., 2018), with permission of Springer Nature].

The AuNPs exhibited better antiviral activities when permitted to interrelate with the virus. The preparation at the absorption of 2 or 4 ppm was further active for hindering the viral access. AuNPs served as a virus entrance inhibitor and as a deactivating agent. Chiodo et al. (2014) described carbohydrate-doped AuNPs which are conjugated along with nucleoside opposite transcriptase inhibitors called lamivudine. AuNPs reserved HIV viral duplication *in-vitro* parallel to drugs. The distribution of applied-medicine from AuNPs repressed viral duplication thus dismissing the development of viral DNA. Currently, there are inadequate investigation studies on AuNPs applications for herpes infections. Sarid et al. (2014) designed sulfonate-covered hydrophilic AuNPs for the inhibition of herpes infections. AuNPs interrelated with the virus through preventing virus-related accessory and dispersion into the cells, thus avoiding infections. Nanotechnology's applications for the cancer cell treatment are based on primary tumor exposure and the analysis by Nano-devices accomplished of selective point and the distribution of chemotherapeutic treatments to definite tumor position. The notable properties of AuNPs are measured as a substantial source for the analysis of different cancerous cells. Presently, the chemotherapeutic treatment depends on the typical chemo and radiation therapies, with the purpose to execute the cancerous cells. Moreover, these actions may result in numerous adjacent side effects due to mutilation caused to the immediate healthy tissues, interruptions in the analysis (Baram-Pinto et al., 2010). Treating the cancerous cells by employed Nano-range drug deliveries method plays a significant part in disabling the restrictions of predictable action methodologies by offering instantaneous diagnostics (Rashid and Ahmad, 2019). Therefore, a substantial volume of study focuses on the nanocarrier growth of AuNPs and their possible sustainable applications in cancerous biology. The bioinspired AuNPs have become a potential possibility to discover for use in biosensors, targeted drug delivery, photothermal therapies, immunoassays, photoimaging, and photodynamic therapy as shown in Figure 3. Interestingly, in human cancerous and cellular biology, several kinds of AuNPs such as Au nanorods, Au nanocages, Au-stars, nano Au-cubes, and Au nanospheres, have been considered as effective tools (Das et al., 2019; Filli et al., 2019; Pillai, 2019; Shrivastava et al., 2019). Despite all these assistances, the biocompatibility of AuNPs is a vital factor to be reconsidered for the interpretation of scientific applications. The current role of AuNPs in the treatment and diagnosis of cancer cells, along with the biocompatibility, is discussed in Table 2.

**Table 2:** AuNPs application in cancer cell diagnosis, treatment methods, and its advantages.

**Metallic nanoparticles**	**Particle size (nm)**	**Targeted**	**Cancer cells**	**Methods**	**Method performance**	**References**
AuNPs	15	pAb and mAb anti-CEA antibodies,	MCF7 cells	SERS immunoassay	Different immunoassay method	Chon et al., 2009
AuNPs	35	anti-EGFR	Oral epithelial living Cancerous cell	SPR	Valuable molecular sensors	El-Sayed et al., 2005
AuNPs	20	Heparin	Epithelial	Imaging	therapeutics and Imaging	Shi et al., 2007
AuNPs	-	folic acid, fluorescein isothiocyanate	Liver cells-imaging	Imaging	Possible CT imaging	Sun et al., 2009
AuNPs	15	Functionalized shiny carbon electrodes	Lung and liver cancer,	Electrochemical and contact-based angle measurements	Fast identification and vastly sensitive exposure for cancerous cell	He et al., 2009
AuNPs	30	ENO1 antibodies	Lung cancer	Electrochemical immune sensor	Measurable tests protein and cancerous biomarkers	Ho et al., 2010
AuNPs	100	Anticarcinogen-embryonic antibodies	Cancer	ELISA	Premature diagnosis of cancer	Zhou et al., 2011
AuNPs	20	kinase C (PKC-alpha) peptides	Breast cancer	GNP- colorimetric assay	Preliminary screening throughout cancer diagnosis	Raghavendra et al., 2014
AuNPs	15	anti-CA15-3-HRP antibodies	Breast cancer	ELISA	Detection of cancerous cells	Ambrosi et al., 2009
AuNPs	90	EGF-ligand and tag Raman receptors	Flow tumors	SERS	Novel clinical device for managing of patients	Wang et al., 2011
AuNPs	25	anti-EGFR	Paracervical cancer	Illumination microscopy,	Influential device for detecting cellular, molecular variations	Rahman et al., 2005
AuNPs	15	Single-chain variable part	Oral cancer	SERS	Saliva-assay for initial analysis of oral Cancerous cells	Kah et al., 2007
AuNPs	60	anti-EGFR	Head-and-neck	SERS	Optical and EM enquiries for tumor detection	Conde et al., 2012
AuNPs	45	Her-2/neu antigen	Prostate cancer detection	Difference photoacoustic imaging	Filmic device for molecular and structural information	Agarwal et al., 2007
AuNPs	15	F19 monoclonal antibodies	Pancreatic adenocarcinoma	Light sprinkling, size prohibiting chromatography	Labeling method	Eck et al., 2008
AuNPs	20	Aptamer	Leukemia and lymphoma	Spectroscopic method	Primary and precise exposure of cancer	Medley et al., 2008

*EGF, endothelial growth factor; SPR, surface plasmon resonance; EGFR, endothelial growth factor receptor; HER, human epidermal growth factor receptor; ScFv: single chain variable fragment; CT, computed tomography; ELISA, enzyme linked immune sorbant assay; SERS, surface enhance Raman scattering; ENO1, alpha-enolase; SCCHN: squamous cell carcinoma of the head and neck; PC-3, prostate cancer cells; pAb, polyclonal antibody; mAb, monoclonal antibody; PEG, poly ethylene glycol; CEA, carcinoembryonic antigen*.

### GNPs as an Inhibitor of HIV, Cancer Vaccines

The immune system of our body is capable of removing some viral infections itself. However, some of the infections such as herpes, hepatitis, HIV, are long-lasting, persistent, and cannot be removed. AuNPs are used as a delivery scheme for the improved effectiveness of anti-HIV activity. The free AuNPs are unproductive against HIV infection. Kesarkar et al. (2012) found that stabilized AuNPs in amino acid L-cysteine resulted in the enhanced azidothymidine delivery against the HIV-1Ba-L virus *in-vitro*. AuNPs are employed for the anti-HIV activities at the initial phase of virus-related replication. The anti-HIV activities of AuNPs are due to poly-anionic shallow that allows the binding with positively charged amino acids at the binding position of glycoprotein gp120, the mechanism of inhibition takes place and the AuNPs block the reverse transcriptase enzyme of HIV-1 proteins. Garrido et al. (2015) studied conjugated AuNPs with better HIV activities. Free AuNPs did not show anti-HIV activities. Kesarkar et al. (2012) also studied the coated AuNPs through polyethylene glycol.

Rich and Miszka (2000) studied the role of oligiomannoside as an inhibitor of gp120 binding to 2G12, using the Surface plasmonon Resonance (SPR) technique for molecular interactions. SPR makes a significant contribution to understand the molecular basis of the HIV life-cycle; mannose-GNPs act as an inhibitor of gp120 binding to DC-SIGN (Martinez-A' vila et al., 2009). Marradi et al. (2011) demonstrated the ability of mannose-GNPs to bind 2G12 and to inhibit 2G12/gp120 binding. The benefit of multivalent therapeutics of gold NPs transform a feeble binding and biologically inactive small molecule into a multivalent conjugate that acts precisely against the HIV-1 fusion to human T-Cells (Bowman et al., 2008; Parry et al., 2013). The high degree of multivalences and the easily controllable size of GNPs provides a great significance for biological application. The carbohydrate-coated GNPs have an advantage of non-toxic environments (Goodman et al., 2004; Ojeda et al., 2007). Brinãs et al. (2012) demonstrated the design and synthesis of AuNPs with multifunctionality that were loaded with tumor-associated glycopeptide antigens and acted as a potential cancer vaccine. Cai et al. (2016) prepared the anticancer vaccines from AuNPs-based vaccines against the tumor-associated form of the mucin-1 glycoprotein.

## Silver Nanoparticles

### A Remedy for Antibacterial and Viral Diseases

The researchers have stated the importance of AgNPs in the treatment of different diseases as shown in Table 3. AgNPs interrelate with microbes and discharge the silver ion in the de-activation of cellular-based enzymes, delayed membrane penetrability. Several scholars have highlighted the antibacterial activities through AgNPs (Adetunji, 2019; Tyagi and Kumar, 2019), mode of action against bacterial germ and cellular-based antimicrobial activities.

**Table 3:** AgNPs function in the treatment of infections.

**Nanoparticles**	**Nature of infection**	**Therapeutic conclusion**	**References**
AgNPs	Malaria	Prevention of growth of *P. falciparum*	Murugan et al., 2016
	Leishmaniasis	Limitation of proliferation and metabolic activities of promastigotes.	Zahir et al., 2015
	Helminth infections	Improved anthelmintic activities in contradiction of worm	Nadhman et al., 2016
	HIV	Inhibition of CD4-based virion binding, fusion, and infection	Elechiguerra et al., 2005
	Herpes	Virus duplication was reserved	Hu et al., 2014
	Herpes	Reserve of viral access into cell and inhibition of ensuing infection	Baram-Pinto et al., 2009
	Hepatitis	Interface with the HBV viral units subsequent in reduction of production of HBV RNA and extra-cellular	Lara et al., 2011
	Influenza	Active against influenza viruses	Rafiei et al., 2018
	Influenza	Active against influenza A virus	Taubenberger and Morens, 2008
AgNPs synthesize through biological approaches by using fungi, algae, bacteria and virus	Bacterial infection	Excellent antibacterial activities	Shirley et al., 2010
AgNPs and amoxicillin, azithromycin, clarithromycin, linezolid, or vancomycin	Bacterial infection	Better synergistic antibacterial effects against methicillin-resistant	Akram et al., 2016
AgNPs and gentamicin and penicillin	Bacterial infection	Exceptional antibacterial properties against animal bacterial infections,	Smekalova et al., 2016
AgNPs mixture with lactam; quinolone; aminoglycoside and polykeptide	Bacterial infection	Active against drug-resistant bacteria *Salmonella typhimurium*.	Deng et al., 2016)
AgNPs mixture with amoxicillin	Bacterial infection	Excellent synergistic effects against *E. coli*	Li et al., 2005
AgNPs shared with polymyxin B and rifampicin	Bacterial infection	High synergistic effects against Acinetobacter, baumannii infection.	Srinivasan et al., 2006
AgNPs in urinary catheter	Bacterial infection	Active NPs against bacteria that are answerable for urinary tract infections.	Wan et al., 2016
Nanowires of AgNPs	Bacterial infection	Nano-cube AgNPs showed the highest antibacterial activities.	Romaniuk and Cegelski, 2015
Hexagonal and nanoplates AgNPs	Bacterial infection	Hexagonal-shaped AgNPs were actual against *S. aureus* and *E. coli*	Hong et al., 2016
Rod-shaped AgNPs	Bacterial infection	Triangular shaped revealed high antibacterial activities against *E. coli*	Pal et al., 2007

AgNPs are also active significant therapeutics for HIV infection treatment (Lara et al., 2010) defined the status of AgNPs as an anti-HIV at the initial phase of viral duplication and later phases of the HIV-life cycle. AgNPs serve through the binding process to gp120, then subsequent inhibition of CD4-reliant on binding, blend, and infection, though the antiviral approach of AgNPs is not entirely understood yet. Herpes disease is a sickness produced by herpes virus HSV-1 and HSV-2. The HSV-2 is related to sexually spread diseases. AgNPs are very active to prevent the development of this virus at both pre and post stages (Sun et al., 2005; Marambio-Jones and Hoek, 2010). Contact between AgNPs and HSV-2 has caused a substantial decrease in the posterity of viruses with feeble cytotoxicity *in-vitro*. AgNPs bond with the glycoprotein membrane of HSV-2, resulting in the contact that delayed virus internalization. This is due to the interfacial glycoprotein and their ability to act as a receptor. Tannic acid has improved the AgNPs which could reduce the HSV-2 contamination both *in-vivo* and *in-vitro* (Elechiguerra et al., 2005; Huang et al., 2018; Verma and Maheshwari, 2019). The antiviral activities of AgNPs were designed through the fractional particle size and dose formulation. AgNPs affect hepatitis B which has been described earlier with the particle size of 10–50 nm. The *in-vitro* anti-HBV assessment of these units on the Hep AD38 cell line exposed that the AgNPs have condensed the extra-cellular HBV. AgNPs interacted with the HBV virus-related particles and caused a reduction of HBV, RNA production and extracellular virus. Helminth infections are measured by tropical desert diseases (Bai et al., 2018; Khatami et al., 2018). The helminth is dependent on worms which are like invertebrate, extended, smoothed or flatforms. AgNPs are also control the infection caused by helminth. AgNPs are very active in the bio-treatment of several types of contaminations such as bacterial, viral and parasitic and work as active meditators (Alam et al., 2017). The biosynthesized self-dispersed AgNPs prepared from the extract of *P. peruviana* acted as a chemical sensor for the dl-alanine amino acid (Rashid and Sabir, 2014; Rashid et al., 2016).

## Palladium Nanoparticles

Pd is a very precious metal with extraordinary catalytic, powerful mechanical, and electroanalytical properties. Pd nano-based structures have been developed as self-therapeutics, with proven anti-bacterial and cytotoxic pharmacological activity. Adams et al. (2014) studied the PdNPs' size-based antibacterial activities. The PdNPs showed high growth prevention against *S. aureus*, as compared to another bacterial species called *E. coli*, the importance of PdNPs is valuable against the anti-bacterial agent, particularly for gram-positive microbes. Sachse et al. (2013) described that the PdNPs fabricated on mesoporous silica nanomaterials showed moderately higher cytotoxic activities against human cancer cell lines. Fascinatingly, these fusion materials also exhibited catalytic activities and were employed for carbon-carbon bonds through Suzuki–Miyaura cross-coupling amongst minor molecules. If PdNPs are the stimulating agent as a self-therapeutics, the additional research is required to clarify their mechanism before they could be used appropriately as a nanomedicines (Dumas and Couvreur, 2015). Taking the benefit of metallic catalytic and optical features, the significant therapeutic-based applications have involved three courses of Pd nanostructures as shown in Figure 3.

Metallic nanostructures facilitated the photothermal (PT) therapies that have invited wide attention recently. The nanotechnology relies on the photon energy absorption, through after the absorption of energy tumor-confined nanostructures that kill the nearby cells. For this persistence, the utilization of near-infrared (NIR) light is the basis usually desired. Au nanostructures are extensively applied in the PT therapy mediators due to their higher destruction co-efficient in the NIR area, higher PT adaptation efficiency and adequate biocompatibility (Xiao et al., 2014; Dumas and Couvreur, 2015; Wei et al., 2015). The PdNPs were not measured as an effective PT mediators until Huang et al. (2011b); and Stone et al. (2016), with an ultra-thin range of 1.8 nm, and hexagonal PdNPs-based sheets that show the well-organized size-dependent and tenable absorption points in the NIR zone with resourceful PT conversion. The Nano-sheets of 41 nm range were employed to kill the liver-based cancer cells and irradiated with 808 nm laser light that shows the higher biocompatibility. Interestingly, these nano-sheets display healthier photo-stability when associated with Au/Ag nanostructures (Huang and Juang, 2011; Mendoza-Pérez and Guisbiers, 2019).

## Mechanism of Toxicity

The physical and chemical reactivity can be a source of formation for free radicals or reactive oxygen species (Gahlawat and Choudhury, 2019; Valavanidis, 2019). The superoxide anions-based radical, and hydroxyl-based radicals, directly or indirectly affect the oxidation of enzymatic pathways, resulting in a form of oxidative stress. The oxidative stress is caused by any of following the reason (Fard et al., 2015)

- Oxidant produced properties of NPs themselves in addition to their ability to produce reactive oxygen species (ROS) as a fragment of cells response to NPs
- Noble metal-based NPs or metal impurities employed as catalysts throughout the generation of non-metal NPs.
- Free radical intermediates exist on surfaces of NPs that are the most reactive.
- Redox vigorous clusters from NPs functionalization (Montalvo-Quiros and Luque-Garcia, 2019).

### Cytotoxicity of Au/Ag/Pd Nanoparticles

AuNPs are the most capable and precious inorganic-based NPs which have attracted a lot of technical and scientific attention due to the presence of significant synthesis methods, better biochemical constancy, and the outstanding optical features. These exclusive topographies of AuNPs refer to them as an interesting approach for cancerous cell diagnosis and action. The *in-vitro* studies have revealed that the AuNPs are less toxic for cellular systems. Cytotoxicity assessment of AuNPs is important because of the wide-ranging variety-based application of AuNPs in the medical field (Khlebtsov and Dykman, 2011; Heo et al., 2012; Fard et al., 2015). In the literature, it has been demonstrated that some inorganic NPs are almost intolerant to the toxicity. The cytotoxicity depends on shape, size and adjacent ligands. Some studies showed that globular AuNPs are appropriate for medical applications (Taylor et al., 2012). The cytotoxicity effect from 5 to 15 nm AuNPs, *in-vitro* on Balb/3T3 mouse fibro-blasts have been examined. To comprehend the experiential alterations in different sized AuNP's cytotoxicity, Coradeghini et al. (2013) observed the acceptance and outside relation of cellular circulation of NPs. There are toxic effects for cellular treatment with 5 nm AuNPs, but no toxic effect is exposed to Balb/3T3. This reflection occurs due to the quantity of 5 nm AuNPs taken-up through cellular evaluation of the larger particle size. Similarly, in the case of AgNPs, antimicrobial characteristics of AgNPs are the source of NP usage in a wide variety of customer products including cosmetics, microchip technology, domestic appliances, fabric material, and nutrition products. Currently, AgNPs have been employed in biomedical fields like drug delivery, manipulative biosensing, and bioimaging, etc. Therefore, the toxicity assessment is of significant importance that is measured in many applications for medical resolutions (Chaloupka et al., 2010). The cytotoxicity of AgNPs is connected to the relaxed oxidation of Ag+ ions which are toxic for biotic systems and the cell components. Gliga et al. (2014) found that the AgNPs in an aqueous culture is toxic and it is associated with the bulk AgNPs. Recently, whether the toxicity of AgNPs depended on coating and size was examined. The toxicity of NPs of 10 nm size was not dependent on the surface coating. In distinction, AgNPs produce a rise in the inclusive DNA injury after 24 h that recommends the self-regulating pieces of machinery for the toxicity and DNA harm. Though there was no enlarged intracellular ROS production, consequently, the toxicity is examined which is associated with the rate of intracellular release of Ag (Batchelor-McAuley et al., 2014). Contact with amino and thiol groups of molecules and the presence of toxicity on the cell components is a consequence of Ag release. Hence, AgNPs with increased Ag release is more deadly (Yang et al., 2011; Wang et al., 2012). PdNPs are usually employed in catalytic applications, where it can be discharged into the ecology through abrasion. These PdNPs may be moved into the surface of the water, with no data approximating their toxicity in the water system. Gurunathan et al. (2019) studied the particle size growth of PdNPs; with hydrodynamic 70 nm particles in media culture with the adjustable ionic asset. The PdNPs agglomerated extra fast with cumulative ionic strength, it produced a solitary marginal influence in an equally assessed species after 96 h of contact. Considering the lower ecological concentration of Pd in the superficial water that is usually ng/L, however, this results in a lower marine hazard in response to the PdNPs. Despite the inclusive applications of NPs in several areas of science, there are frequent remarks about the antagonistic effects of NPs on the biotic systems and cell partitions. Furthermore, the physicochemical activities, toxic ions production, structure of fibrous, higher surface charges, radical species generation, all result in toxicity through Ag/Au/Pd NPs (Leso et al., 2019). The condition of *in-vivo*/*in-vitro* assessments are employed to evaluate the harmfulness of nanomaterials. The *in-vitro* assessments have acknowledged the extra considerations associated with *in-vivo*, due to closer, non-suitable, and the absence of ethical issues (Shubha et al., 2019).

### Quantitative Toxicity Analysis

Factors such as surface modification, size, concentration and the dose of NPs contributes to the bio distribution and toxicity under *in-vivo* and *in-vitro* conditions. The general sites of accumulation and biodistribution for AuNPs and AgNPs are liver, spleen (Van der Zande et al., 2012; Cui et al., 2018). The NPs primarily affect the mononuclear phagocyte system of these organs. The extra dose of AgNPs are deposited and stored in heart, lungs and kidneys whereas AuNPs are exclusively found in the liver. Another preferential site of AgNPs is blood and fecal matter. Yang et al. (2017) reported the toxicity and the biological distribution of AuNPs and AgNPs by intravenous administration of 11.4–13.3 mg/kg in mice. The administration of 5, 10, 45, mg/kg of AgNPs resulted in a decrease of body weight as examined by Zhang et al. (2013). However, the exposure to these NPs through oral administration or inhalation affect the growth of the organs. In the toxicity studies, the dose of the AuNPs/AgNPs is very significant for the biodistribution of these NPs in different tissues and body organs (Yang et al., 2017; Ma et al., 2020; Rodriguez-Garraus et al., 2020). To prevent the accumulation, there is no fully proven mechanism in the literature that discuss the removal of such NPs from the affected organs.

## Future Prospect and the Concluding Remarks

Metal-based NPs have been suggested to overcome the limitations of drug resistance. These metallic Au/Ag/Pd NPs show positive cellular interactions with bio-molecules, both inside and on the surface of cells. They can also be applied by presenting designated biotic moieties through definite binding activities to a particular target and help in promoting their healing efficiency. Their outstanding cell contact has been used by many scholars to grow therapeutics for different viruses and for infectious diseases. Metallic NPs have been suggested to defeat co-viral contamination to increase the effectiveness of anti-viral medications, valuable for prophylactic, healing, diagnosis, photo thermal therapy, photoacoustic imaging, and laser therapy for cancer cells. Furthermore, there is insufficient information, demonstrating that there is a critical need for further research in designing of metallic-based NPs for viral infections treatment (Ngwa et al., 2014). Despite all applications of Au/Pd/Ag NPs, the most famous application is in the detection and treatment of tumor cancer cells, molecular bio-imaging, complex investigation, and immunoassay. Unfortunately, still, there is no absolute and complete treatment, protective vaccine or suitable analysis for cancer cells and AIDS. The AIDS/HIV disease exists in humans to an extent that is measured to be at the epidemic level. Moreover, in spite of the fast progress in the field, new cancerous cell cases have been silently cumulative. Consequently, to promote the use of NPs widely in the biotic field, novel preparation, fabrication, and characterization approaches are desirable for the highly emerging and progressive field of nanotechnology (Bracey et al., 2009). To progress with biogenic NPs in diverse directions and their capability to modify the physical/chemical features, the NPs must possess the higher biocompatibility, high water dispersion, colloidal and physiological stability, making them more effective for treatment. However, sincere efforts are needed to minimize the toxicity level and to recover the non-invasive property so they can absorb the near-infra-red (NIR) light. The plasmonic property of NPs must not be compromised, thus, they can be effectively used in photothermal therapy and the imaging of tumor cells.

They can be better applied to make a more progressive and healthy binding affinity with several biomolecules and targeted drugs for treatment, detection of diseases such as cancer and HIV. In the future, it could be possible to employ these properties and to promote the green chemistry approach for the preparation of smart resources for diagnosis and their applications (Chesney, 2006). NPs hold excessive potential due to the anti-bacterial, anti-fungal, anti-viral, and anti-inflammatory characteristics, while the current study has mentioned the different osteoinductive features as well. The biological mechanisms and the relations behind these kinds of features are not entirely understood. For example, the association of AgNPs, shape, size and especially their biotic features and the cytotoxicity is not visibly studied. Therefore, additional study of AgNPs usage is needed for better outcomes. There is a persistent need to explain the concerning mechanism and the level of toxicity before the extensive biomedical application (Shubha et al., 2019). Despite their valuable chemical and physical features, PdNPs have been preoccupied with Nano-therapeutics collections. They also require an entire understanding of involved factors such as biodistribution and pharmacokinetics, metabolism mechanism, long-lasting toxicity, and mechanism of pharmacological activities (Barrabés and Sá, 2011). The interactions of nanostructures with cellular and biotic ecological is another matter that deserves a profound study, particularly the pre- and post-modification of NPs. The development of nanotechnology based on PdNPs with significant therapeutic characteristics is new, demonstrating only a few publications. Yet, their exclusive properties and significantly lower cytotoxicity means they are emerging key players in the Nano-medical field. Also, some innovative applications will be revealed through the usage of their optical properties for analysis, or extremely good-looking open-ended determinations (Lazarević et al., 2017). The several surfaces of Pd nanostructures provide modest systems that are able to accomplish many collective effects. Finally, overall, there is a crucial need to explore the toxicological properties and pharmaco-kinetics of metallic-based complexes. The medicines derived from organic compound show the drug resistance whereas metallic NPs have no such issues. There is no hesitation that these NPs are the most promising and emerging in the field of applied therapeutics.

## Author Contributions

SY and RR wrote the draft of the article. MR designed the outline and revised the draft. RA did the final corrections in the manuscript.

## Conflict of Interest

The authors declare that the research was conducted in the absence of any commercial or financial relationships that could be construed as a potential conflict of interest.
